# Das gute Leben: Gesellschaft, Politik und die Praxis des hohen Alters

**DOI:** 10.1007/s00391-021-01979-4

**Published:** 2021-10-08

**Authors:** Christiane Woopen, Michael Wagner, Susanne Zank

**Affiliations:** 1 Forschungsstelle Ethik, Uniklinik Köln; ceres – Cologne Center for Ethics, Rights, Economics, and Social Sciences of Health, Universität zu Köln, Albertus-Magnus-Platz, 50923 Köln, Deutschland; 2 Institut für Soziologie und Sozialpsychologie, Universität zu Köln, Albertus-Magnus-Platz, 50923 Köln, Deutschland; 3 Humanwissenschaftliche Fakultät, Rehabilitationswissenschaftliche Gerontologie, Universität zu Köln, Albertus-Magnus-Platz, 50923 Köln, Deutschland

**Keywords:** Lebensqualität, Ethik, Wohlbefinden, Gelingendes Leben, Alterspolitik, Quality of life, Ethics, Well-being, Flourishing life, Age policy

## Abstract

Im abschließenden Beitrag stellen die Autor*innen das Konzept „gelingende Lebensführung“ in den Mittelpunkt. Die Kernergebnisse der vorhergehenden Beiträge werden zusammengefasst und im Rahmen einer kritischen Diskussion in dieses Konzept eingeordnet. Vor dem Hintergrund werden Empfehlungen für die politische und gesellschaftliche Gestaltung von Rahmenbedingungen formuliert, die einer gelingenden Lebensführung im sehr hohen Alter dienlich sind und sie fördern.

## Abstract (en)

In the final article the authors focus on the concept of “flourishing life”. The core results of the preceding articles are summarized and placed in this concept in the context of a critical discussion. Against the background, recommendations are formulated for the political and societal shaping of framework conditions, which are conducive for and promote a good life in very old age.

## Zum Verhältnis von Lebensqualität und gelingender Lebensführung

Fragt man nach der *Qualität* und nicht nach der Quantität des Lebens, so fragt man danach, welche Eigenschaften das Leben hat, und ob es ein gutes oder ein schlechtes Leben ist. Diese Frage kann man aus unterschiedlichen *Perspektiven* betrachten. So kann das betreffende Individuum, um dessen Leben es geht, selbst befragt werden, wie es seine Lebensqualität einschätzt; es kann aber auch eine andere Person angeben, wie sie die Lebensqualität des Betreffenden einschätzt, und es kann ganz allgemein darüber nachgedacht werden, welche Bedingungen erfüllt sein müssen, damit man von einem qualitativ guten Leben sprechen kann. Zudem kann die Qualität des Lebens in unterschiedlichen *Hinsichten* wie etwa bezüglich der Gesundheit, des sozialen Netzwerks oder der Wohnbedingungen beurteilt werden. Nicht zuletzt kann das Wort „gut“ ganz Unterschiedliches bedeuten, zum Beispiel problemlos, altersgemäß, erfolgreich oder moralisch gut.

Die Frage nach der Qualität des Lebens kann auf unterschiedlichen *Ebenen* gestellt werden: Es kann auf einer deskriptiven Ebene beschrieben werden, wie Menschen tatsächlich leben; es kann auf einer evaluativen Ebene bewertet werden, wie sie leben, und es können auf der normativen Ebene Regeln und Maßstäbe dafür angesetzt werden, wie Menschen leben sollten. Alle diese Ebenen haben ihre jeweilige Rechtfertigung. Auf allen diesen Ebenen ist eine Dimension einschlägig, die üblicherweise nicht ausdrücklich thematisiert wird, nämlich die ethische.

*Warum* interessiert die Frage nach der Qualität des Lebens? Weil es – so der Fokus dieses Beitrags – perspektivisch stets um *das gute Leben* im ethischen Verständnis des Wortes „gut“ geht. Die Frage nach der Lebensqualität zielt mehr oder weniger explizit stets darauf, ob die betreffende Person ein Leben führen kann, das sie als sinnvoll betrachtet, das für sie ein gutes im Sinne eines gelingenden Lebens ist. Wenn wir alle Hinsichten, die wir bei der Lebensqualität untersuchen und als relevant erachten, wie beispielsweise die Gesundheit, die finanziellen Ressourcen oder die Freizeitaktivitäten, daraufhin befragen, *warum* sie uns interessieren, stoßen wir über mehr oder weniger zahlreiche Zwischenantworten, die immer wieder auf ihr „Warum“ hinterfragt werden, auf eine letzte Antwort, die in der einen oder anderen Weise mit einem glücklichen und als sinn- und damit wertvoll empfundenen Leben zusammenhängt und bei der es nicht mehr sinnvoll ist, weiter nach dem „Warum“ zu fragen. Warum möchten Menschen ein gelingendes, ein sinnvolles Leben führen? Weil das gelingende Leben um seiner selbst willen als etwas Gutes und Wünschenswertes erachtet wird und nicht darüber hinausgehenden Zwecken dient [2]. Ein glückliches und gelingendes Leben im hier gemeinten Sinne ist dementsprechend nicht deckungsgleich mit dem psychologischen Konstrukt des subjektiven Wohlbefindens, das üblicherweise die ethische Komponente nicht ins Zentrum stellt. Das Konzept eines gelingenden Leben setzt im hier gemeinten Sinne zudem keine spezifische ethische Theorie voraus, sondern nur die in vielen ethischen Theorien in der einen oder anderen Weise getroffene Annahme, dass ein Mensch sein Leben dann als gelingend empfindet, wenn er diejenigen seiner Eigenschaften entfalten und Werte sowie Überzeugungen leben kann, die ihm besonders wichtig sind.

Aus dem hier zugrunde gelegten Begriffsverständnis und diesen Überlegungen folgt, dass jemand seine summarisch betrachtete Lebensqualität dann nicht als schlecht erachten kann, wenn er aus seiner Sicht ein gelingendes Leben führt; er kann gleichwohl ein eingeschränktes subjektives Wohlbefinden haben, sich dessen bewusst sein, dass seine Lebensqualität von außen als schlecht beurteilt wird oder gängigen Vorstellungen seiner Zeit und seiner Kultur zu einem guten Leben nicht entspricht, und er kann auch mit bestimmten Aspekten seines Lebens unzufrieden sein. Ebenso kann er seine Lebensqualität insgesamt nicht persönlich für gut halten, wenn er sein Leben als sinnlos empfindet. Gleichwohl kann er sich in bestimmten Hinsichten wohlfühlen und zufrieden sein. Aus der Außensicht kann sich das anders darstellen: Man kann die Lebensqualität eines Menschen je nach zugrunde gelegten Maßstäben aus der Außenperspektive für gut halten, obwohl der Betreffende selbst sein Leben für sinnlos hält – und umgekehrt. In unterschiedlichen Varianten und Kontexten werden derartige Inkongruenzen auch als Lebensqualitäts‑, Wohlbefindens- oder Zufriedenheitsparadox bezeichnet.

Damit ist freilich noch nicht die Frage beantwortet, *was im Detail* für ein konkretes Individuum oder eine gesellschaftliche Gruppe ein gutes, glückliches, gelingendes und als sinnvoll empfundenes Leben bedeutet. Die Antworten darauf können in der modernen und freiheitlich verfassten Gesellschaft sehr unterschiedlich ausfallen und zeigen sich in ganz unterschiedlichen Arten zu leben. Ist der eine glücklich, wenn er oft viele Freunde sieht, ist es dem anderen wichtiger, viel Ruhe zu haben und eine intensive Freundschaft zu einer einzelnen Person oder nur wenigen Freunden zu pflegen. Es gibt glückliche Menschen mit geringen finanziellen Ressourcen und unglückliche reiche Menschen. Aus Ressourcen, welcher Art auch immer, kann man – eine gewisse Mindestausstattung vorausgesetzt – nicht darauf schließen, wie sehr ein Mensch sein Leben als ein gutes Leben betrachtet. Ganz im Gegenteil kann eine zu starke Orientierung an der Steigerung oder Optimierung von Ressourcen einem gelingenden Leben sogar im Wege stehen [14].

Auch *ethische Theorien* unterscheiden sich in der ihnen jeweils zugrunde liegenden Vorstellung von einem guten Handeln und einem im Handeln gelingenden Leben [11]. Grob skizziert ist für Tugendethiker dasjenige Handeln gut, das aus einer tugendhaften Haltung erwächst [2]; Utilitaristen geht es um das größte Glück für die größtmögliche Zahl, und sie bemessen es anhand der Folgen des Handelns, die insbesondere danach beurteilt werden, ob die Summe der je individuellen Lust [3] oder die Erfüllung der jeweiligen Präferenzen [17] maximiert wird; für den Deontologen ist das gute Handeln dasjenige, das der Vernunft und der Pflicht entspricht, und dessen Maximen im Einklang mit dem kategorischen Imperativ stehen [9].

Es wird deutlich, dass weder auf der konkreten individuellen Ebene noch auf der Ebene theoretischer Zugänge Einigkeit darüber besteht, was ein gutes Leben für eine konkrete Person präzise ausmacht. Wie aber misst man dann Lebensqualität? Es wird deutlich, dass Forschung, die sich mit ihrer Messung befasst, immer eine mehr oder weniger explizite Vorstellung von einem guten Leben voraussetzt, auf deren Grundlage bestimmt wird, *welche Fragen* gestellt, *wem* die Fragen gestellt, welche* Erhebungsmethoden *verwendet, wie die *Ergebnisse* kontextualisiert und interpretiert, welche *Schlussfolgerungen* daraus gezogen und vielleicht sogar welche (politischen) *Handlungsempfehlungen* gegeben werden. Es gibt – so die hier vertretene These – *keine Lebensqualitätsforschung, die frei wäre von gehaltvollen ethischen Annahmen und Werten* [10].

Vor diesem Hintergrund sind auch *Konzepte der Alternsforschung* zu diskutieren, die die Diskussion prägen. Dazu gehört etwa das Konzept des „erfolgreichen Alterns“ („successful aging“), which „is defined by an autonomous, generative, active, or productive behavior by using respective educational, social, infrastructural, technical, or economic resources“ [6]. Ein nur minimale Ressourcen verwendendes kontemplatives Leben eines zurückgezogenen Mönches etwa erscheint vor diesem Hintergrund kein Beispiel für erfolgreiches Altern zu sein. Schon der Begriff „erfolgreich“ transportiert die grundlegende Wertentscheidung, dass Erfolg eine wünschenswerte Zielgröße oder zumindest ein Maßstab sei. Somit ist Erfolg notwendigerweise ein normatives Konzept. Doch auch der Begriff „Erfolg“ ist konkretisierungsbedürftig. Was konkret zählt als Erfolg? Wer definiert und entscheidet, ob die Lebensführung erfolgreich ist? Hat etwa jemand, der resilient mit persönlichen Niederlagen im Leben umgehen kann, eine schlechte oder eine gute Lebensqualität? Tesch-Römer und Wahl plädieren vor diesem Hintergrund für eine Weiterentwicklung des traditionellen Konzeptes von Rowe and Kahn, um angesichts einer zunehmend steigenden Lebenserwartung auch Behinderungen und Pflegebedarf in ein Konzept von „successful aging“ zu integrieren, wobei sie Autonomie und Wohlbefinden als wesentliche Elemente von wünschenswerten Lebensbedingungen voraussetzen [18]. Dieses Modell ist zwar integrativ und bietet wertvolle Ansatzpunkte für gesellschaftliches und politisches Handeln; die grundlegende ethische Problematisierung der Gleichsetzung von Erfolg und guter Lebensqualität vermag es gleichwohl nicht zu beantworten.

Im Rahmen des Forschungsprojektes NRW80+ wurde mit dem *CHAPO-Modell* ein Rahmenkonzept vorgelegt, das für unterschiedliche Auffassungen von einem gelingenden Leben offen ist und einen integrativen Ansatz der Forschung zur Lebensqualität ermöglicht [6, 20]. Das CHAPO-Modell berücksichtigt sowohl die subjektiven Auffassungen der befragten Personen (im Falle fehlender Befragbarkeit mithilfe ihrer VertreterInnen) als auch übergreifende normative Vorstellungen und Alternsbilder. Es werden sowohl unterschiedliche Arten von Ressourcenlagen als auch Ergebnisse im Hinblick auf unterschiedliche Facetten von Lebensqualität einschließlich Wohlbefinden und Zufriedenheit erfasst. Darüber hinaus werden sowohl das Individuum als auch seine Umwelt berücksichtigt. Der Horizont des gelingenden Lebens wird durch einen eigenen Bereich im CHAPO-Modell eigens ausgewiesen.

Im CHAPO-Modell wird vorausgesetzt, dass etliche Konstrukte und ihre Messinstrumente je nach untersuchter Fragestellung in unterschiedliche Bereiche eingeordnet werden können. Das entspricht dem verwandten Modell der vier Lebensqualitäten von Veenhoven [19]. Zudem wird hier vorausgesetzt, dass es allenfalls selten ein unidirektionales Kausalverhältnis von Voraussetzungen und Resultaten gibt, etwa zwischen Umgebungsbedingungen und persönlichen Lebensresultaten. Im Sinne eines komplexen Bedingungsgefüges wird vielmehr davon ausgegangen, dass sich sowohl Umgebungs- als auch persönliche Faktoren, sowohl empirische Gegebenheiten und normative Anforderungen, sowohl Möglichkeiten als auch Resultate kontinuierlich wechselseitig beeinflussen.

## Einordnung ausgewählter Ergebnisse

„Jede Kunst und jede Lehre, desgleichen jede Handlung und jeder Entschluss, scheint ein Gut zu erstreben, weshalb man das Gute treffend als dasjenige bezeichnet hat, wonach alles strebt“ [1]. Das Gute, das erstrebt wird, kann damit auch als Gut verstanden werden, das dem handelnden Menschen so wichtig und so wertvoll ist, dass er sich für dessen Verwirklichung einsetzt – mag es eine banale Alltagshandlung oder ein großer Lebensplan sein. In diesem Sinne kann man das erstrebte Gut auch als Wert beschreiben.

Die Teilnehmer:innen von NRW80+ wurden nach ihren *individuellen Werten* befragt [13]. Von den 10 grundlegenden Werten, die Schwartz in vielfältigen Studien als Set herausgearbeitet und in mehreren Hinsichten geordnet hat, waren den Befragten die folgenden 4 Werte in abnehmender Bedeutung am wichtigsten: Sicherheit, Selbstbestimmung, Tradition und Universalismus. Werte mit einem stärkeren sozialen Fokus spielten insgesamt eine gewichtigere Rolle im Vergleich zu solchen mit persönlichem Fokus. Selbstbestimmung, Benevolenz, Universalismus, Hedonismus und Stimulation waren positiv verbunden sowohl mit dem affektiven Wohlbefinden, das primär emotionale Aspekte und damit den hedonischen Anteil der Wertschätzung des Lebens wiedergibt, als auch mit aktiver Verbundenheit mit dem Leben, die deren eudämonischen Anteil betrifft und für die Befragten vergleichsweise größer ist. Dies zeigte, dass nur Werte mit einer Orientierung an Wachstum statt Bewahrung für das affektive Wohlbefinden bedeutsam sind. Dieselbe Korrelation mit der Wertschätzung des eigenen Lebens galt für die im Rahmen der Erhebung zur Spiritualität angegebene Verbundenheit mit der Natur und mit anderen Menschen. Zudem war eine transzendentale Verbundenheit bedeutsam, wohingegen religiöse Institutionen und Praktiken von untergeordneter Wichtigkeit waren.

Welch große Bedeutung für das Autonomieempfinden die Möglichkeit hat, nach den eigenen Präferenzen und Vorstellungen leben zu können, zeigt auch die Analyse der Diskrepanzen zwischen den Dispositionen der Befragten und der gelebten Alltagspraxis in ihrer Abhängigkeit von Pflegebedürftigkeit, Multimorbidität und privater Pflegetätigkeit [5].

Summarisch kann also festgehalten werden, dass Entfaltungsmöglichkeiten im Rahmen einer Verbundenheit mit der menschlichen und nichtmenschlichen Umgebung sowie die Möglichkeit, nach den eigenen Vorstellungen leben zu können, für hochaltrige Menschen eine große Bedeutung für ihre Lebensqualität haben. Im CHAPO-Modell können individuelle Werte konzeptionell als individuelle normative Lebensmöglichkeiten angesehen werden, aus denen heraus eine Person lebt; sie sind darüber hinaus das Ergebnis eines Lebensweges [8] mit all den individuellen Erfahrungen, die in einem sozialen Kontext gemacht werden, der durch die gegebenen Lebensbedingungen und normativen Standards geprägt wird. Diese normativen Standards finden sich u. a. in Alternsbildern und ihren Stereotypen wieder.

Bei der Untersuchung dieser *Stereotype* in semistrukturierten Interviews mit unterschiedlichen Stakeholdern [12] wurde deutlich, dass die bevorzugten Werte hochaltriger Menschen teilweise im Kontrast zu vorherrschenden Alternsbildern stehen. Aufschlussreiche Unterschiede waren diesbezüglich zwischen solchen Akteuren zu verzeichnen, deren Arbeit in unmittelbarem Kontakt mit hochaltrigen Menschen erfolgt (Gruppe 1), und solchen, bei denen das nicht der Fall ist (Gruppe 2). Etliche Aspekte überschneiden sich zwar, wie etwa die Wahrnehmung vielfältiger Verlusterfahrungen, des Reichtums an Erfahrungen sowie einer gewissen Andersartigkeit im Vergleich zu anderen Altersgruppen. Gleichwohl haben Interviewte der Gruppe 1 in mehreren Hinsichten ein deutlich ausgeprägteres Bewusstsein für die individuellen und sozialen Potenziale hochaltriger Menschen, auch bei gegebenen gesundheitlichen Einschränkungen oder sozialen Abhängigkeiten. Man muss davon ausgehen, dass derartige Stereotype die Lebensbedingungen hochaltriger Menschen und damit auch ihr Selbstbild und ihr Verhalten erheblich prägen, etwa durch die damit zusammenhängende Gestaltung von Angeboten (z. B. Fernsehprogramm, Pflegestrukturen) und gesellschaftliche Diskurse. Schwartz führt treffend aus: Eine Theorie über das Weltall verändert nicht den Lauf der Planeten, aber eine Theorie über die Natur des Menschen ändert den Menschen [16].

Angesichts der erwähnten hohen sozialen Orientierung hochaltriger Menschen ist es bedauerlich, dass sich fast 60 % der Befragten als von der Gesellschaft (überhaupt) nicht gebraucht empfinden; 13,6 % empfinden sich von der Gesellschaft gar als Last behandelt. Dies hing v. a. mit gesundheitsbezogenen Variablen sowie produktiven Aktivitäten (z. B. Mitgliedschaft in einer Organisation oder ehrenamtliche Tätigkeit) zusammen. Auch ergab ein Vergleich von 3 Gruppen der Befragten, die digitale Informations- und Kommunikationsmedien entweder nicht gebrauchten oder ohne bzw. mit Webanbindung gebrauchten, dass diejenigen, die ICT verwendeten und darunter insbesondere diejenigen, die sie mit Internetanbindung nutzten, ein höheres Autonomieempfinden sowie geringere Level von Anomie und Einsamkeit berichteten [15]. Es kann also angenommen werden, dass sich die Lebensqualität hochaltriger Menschen dann verbessern ließe, wenn der Kontakt mit ihnen breiter und Alternsbilder positiver würden. Stereotype können bezogen auf das CHAPO-Modell in diesem Sinne sowohl als normative Lebensmöglichkeiten als auch als Ergebnis gesellschaftlicher Praxis und Institutionen angesehen werden.

Ebenfalls im Sinne einer Quelle und eines Resultates bezüglich der umgebungsbezogenen Lebensqualität kann das *soziale Netzwerk* einer Person betrachtet werden. Bei den Befragten war das Zusammenleben mit einem Partner (35,5 %) mit der Größe des sozialen Netzwerks und beides negativ mit *Einsamkeit* assoziiert. Die Diversität des Netzwerks war bei den ohne Partner Lebenden (59,3 %) am höchsten, während diejenigen, die einen Partner haben, aber mit diesem nicht zusammenleben (5,2 %), das kleinste und am wenigsten diverse soziale Netzwerk hatten.

Der soeben erwähnte Zusammenhang zwischen gesundheitsbezogenen Variablen und der wahrgenommenen gesellschaftlichen Wertschätzung lenkt die Aufmerksamkeit auf die *Gesundheit.* Die gesundheitlichen Bedingungen, unter denen ein Mensch sein Leben führt, beeinflussen als sozial determinierte individuelle Lebensmöglichkeiten erheblich, welches Spektrum an Zielen er mit welchen Erfolgsaussichten verfolgen kann. Sie haben mithin einen großen, wenn auch nicht alles entscheidenden Einfluss auf die Möglichkeiten, ein gelingendes Leben nach den je eigenen Vorstellungen führen zu können [21]. Passend zu dieser ethischen Relevanz von Gesundheit zeigte sich in der Studie NRW80+ ein signifikanter Zusammenhang zwischen *Multimorbidität *und reduzierter Autonomie (ein individueller Wert, der sich bei der Untersuchung als sehr wichtig herausgestellt hatte, s. oben), reduzierter Funktionsfähigkeit und Lebenszufriedenheit, wobei all diese Variablen, bezogen auf das CHAPO-Modell, in ihren jeweiligen Bedingtheiten sowohl als individuelle Ressource als auch als Resultat verstanden werden können. Dies legt einen stärkeren Präventionsansatz nahe, der sich angesichts der Bedeutung für die Funktionsfähigkeit in allen alltäglichen Belangen im Sinne von „health in all policies“ (intersektorielle Gesundheitspolitik) [22] schon früh auf die möglichst lange Erhaltung guter gesundheitlicher Bedingungen richtet.

Im Zusammenhang mit gesundheitlichen Bedingungen ist auch das Konstrukt der *Gebrechlichkeit* („frailty“) zu betrachten [23]. Knapp 19 % der Befragten waren als prägebrechlich, 57 % als gebrechlich und 24 % als robust einzuordnen. Personen, die mit ihrer außerhäuslichen Umgebung nicht verbunden waren, hatten eine höhere Wahrscheinlichkeit, prägebrechlich oder gebrechlich zu sein. Darüber hinaus stand das Wohnen in schlechteren Wohngegenden und in Gemeinden mit mehr als 500.000 Einwohnern in einem signifikanten Zusammenhang mit einer Gebrechlichkeit der Befragten. Im Rahmen eines Health-in-all-policies-Ansatzes sollte dementsprechend ebenfalls die Wohnumgebung einbezogen werden.

Ebenfalls ist die Erfahrung von „*elder abuse*“ (Erfahrung von Gewalt im sozialen Nahraum), die über die Hälfte der Befragten in den letzten 12 Monaten mindestens einmal gemacht haben, negativ mit Autonomie und Lebenszufriedenheit und gehäuft mit depressiven Symptomen und Einsamkeit verbunden [4]. Bei der Interpretation dieser Befunde muss berücksichtigt werden, dass das Konzept „elder abuse“ außerordentlich weit gefasst ist. Als Risikofaktoren wurden Multimorbidität, eine geringere Größe des sozialen Netzwerks und eine größere Aggressivität des Opfers identifiziert. „Elder abuse“ kann im CHAPO-Modell sowohl als Quelle als auch als Resultat, bezogen auf die umgebungsbezogenen Faktoren von Lebensqualität, eingeordnet werden, wohingegen *traumatische Erlebnisse im Zusammenhang mit dem Zweiten Weltkrieg *[7], von denen 42 % der Befragten berichteten, insbesondere zu den umgebungsbezogenen Quellen von Lebensqualität zu zählen sind, die der Betreffende als individuelle Quelle mit durch sein Leben nimmt. Es wurden Zusammenhänge zur Menge von Erkrankungen, zu höherem Bildungsabschluss und höherer Depressivität festgestellt. Kein signifikanter Zusammenhang bestand jedoch mit positivem Affekt und der Wertschätzung des Lebens in der Ausformung als Optimismus und Engagement (im Sinne von Problemlösung sowie Aktivitäten und Zuversicht bezüglich des Erreichens gewünschter Ziele).

## Empfehlungen für die politische und gesellschaftliche Gestaltung von Rahmenbedingungen, die einer gelingenden Lebensführung hochaltriger Menschen dienlich sein können

In diesem Beitrag konnten nur ausgewählte Aspekte unterschiedlicher Elemente von Lebensqualität aufgegriffen werden. Es sollte verdeutlicht werden, dass Lebensqualitätsforschung und die Einordnung sowie Reflexion ihrer Ergebnisse stets von ethisch relevanten Vorannahmen und Kontexten geprägt sind. Etablierte Konstrukte können allenfalls selten ausschließlich einem Bereich eines Lebensqualitätskonzeptes zugeordnet werden.

Vor dem Hintergrund dieser Komplexität können gleichwohl einige Empfehlungen an politische und gesellschaftliche Akteure auf Bundes‑, Länder- und kommunaler Ebene sowie in allen gesellschaftlichen Bereichen wie etwa Wirtschaft, Bildung, Gesundheit, Arbeit und Kultur formuliert werden, deren Umsetzung es nach unseren Erkenntnissen verspräche, die Lebensqualität hochaltriger Menschen in Deutschland und damit ihre Möglichkeiten, ein nach ihren Vorstellungen gelingendes Leben zu führen, perspektivisch zu verbessern. Eine ausschließliche Konzentration auf Ressourcenlagen für hochaltrige Menschen reicht dazu nicht aus. Vielmehr sind von Beginn an Beteiligungsprozesse sowie komplexe Bedingtheiten schon in frühen Lebensaltern von vornherein mitzudenken.

## Fazit für die Praxis

- Hochaltrige Menschen sollten im öffentlichen Bereich verstärkt in die aktive Gestaltung ihrer Lebensbedingungen einbezogen werden und über die ihnen wichtigen Belange mitbestimmen können. Dazu gilt es, partizipative Institutionen und Verfahren (weiter) zu entwickeln.
- Institutionen und gesellschaftliche Praktiken sollten in einer Weise gestärkt werden, dass sie die Entfaltungsmöglichkeiten sowie die Spiritualität im Sinne der Verbundenheit hochaltriger Menschen mit der menschlichen und außermenschlichen Umgebung fördern und dabei auch ihre soziale Orientierung nutzen.
- Nicht zuletzt mit Blick auf die Stärkung der sozialen Verbundenheit als Gegensatz zum Gefühl der Einsamkeit sollten soziale Netzwerke gebildet und gestärkt werden. Dies sollte gerade kommunal die Gestaltung der Wohnumgebung umfassen.
- Der Kontakt von Menschen unterschiedlichen Alters mit hochaltrigen Menschen sollte breit gefördert werden, da dies Alternsbilder positiv beeinflussen und damit Lebensbedingungen verbessern kann. Es sollte schon im jungen Alter (Schule, Ausbildung, Universitäten) eine Sensibilisierung für Stereotype und ihre möglichen Auswirkungen auf die Lebensbedingungen der betreffenden gesellschaftlichen Gruppe erfolgen.
- Angesichts der hohen Relevanz der Gesundheit für die Lebensqualität hochaltriger Menschen mit Blick auf die Möglichkeit einer gelingenden Lebensführung sollte die gesundheitliche Prävention im Sinne eines Health-in-all-policies-Ansatzes (intersektorielle Gesundheitspolitik) schon im frühen Alter und während des gesamten Lebensverlaufs gestärkt werden. Dies umfasst auch lebenslanges Lernen. Es gilt auch für die Wohnumgebung gerade mit Blick auf die Prävention von Gebrechlichkeit.
- Lebenslanges Lernen und die Einbindung in das persönliche soziale Netzwerk sollten sich auch auf die Möglichkeiten der Teilhabe mittels digitaler Technologien beziehen.
- Hohe Aufmerksamkeit sollte aktuellen und vergangenen Erfahrungen hochaltriger Menschen von Gewalt, Trauma und Missbrauch (in einem weiten Verständnis) gelten, insbesondere um negative Effekte auf Depressivität, Autonomie und Lebenszufriedenheit abzuschwächen bzw. zu vermeiden.
- Religiöse Institutionen können in der hohen Bedeutung, die hochaltrige Menschen der Verbundenheit mit Menschen sowie mit der außermenschlichen Umgebung und mit einer den Menschen übersteigenden Größe geben, Anknüpfungspunkte finden, wenn sie ihre Relevanz für hochaltrige Menschen (und wohl auch für Menschen anderer Altersgruppen) angesichts der tiefgreifenden kirchlichen Krise stärken wollen.
